# The human brain somatostatin interactome: SST binds selectively to P-type family ATPases

**DOI:** 10.1371/journal.pone.0217392

**Published:** 2019-05-28

**Authors:** Michael Solarski, Declan Williams, Mohadeseh Mehrabian, Hansen Wang, Holger Wille, Gerold Schmitt-Ulms

**Affiliations:** 1 Tanz Centre for Research in Neurodegenerative Diseases, University of Toronto, Krembil Discovery Centre, Toronto, Ontario, Canada; 2 Department of Biochemistry, University of Alberta, Edmonton, Alberta, Canada; 3 Centre for Prions and Protein Folding Diseases, University of Alberta, Edmonton, Alberta, Canada; 4 Department of Laboratory Medicine & Pathobiology, University of Toronto, Toronto, Ontario, Canada; Universite de Rouen, FRANCE

## Abstract

Somatostatin (SST) is a cyclic peptide that is understood to inhibit the release of hormones and neurotransmitters from a variety of cells by binding to one of five canonical G protein-coupled SST receptors (SSTR1 to SSTR5). Recently, SST was also observed to interact with the amyloid beta (Aβ) peptide and affect its aggregation kinetics, raising the possibility that it may bind other brain proteins. Here we report on an SST interactome analysis that made use of human brain extracts as biological source material and incorporated advanced mass spectrometry workflows for the relative quantitation of SST binding proteins. The analysis revealed SST to predominantly bind several members of the P-type family of ATPases. Subsequent validation experiments confirmed an interaction between SST and the sodium-potassium pump (Na^+^/K^+^-ATPase) and identified a tryptophan residue within SST as critical for binding. Functional analyses in three different cell lines indicated that SST might negatively modulate the K^+^ uptake rate of the Na^+^/K^+^-ATPase.

## Introduction

Somatostatin (SST) is an inhibitory peptide hormone produced by specific cells, including somatostatinergic neurons in several brain regions and somatotropic cells, known as delta cells, in pancreatic islets, the pyloric antrum and the duodenum. SST was initially discovered as a factor that inhibits growth hormone (GH) release from the anterior pituitary [1]. To date, SST is understood to also act as an inhibitor of synaptic transmission in the central nervous system, to regulate insulin and glucagon release from the pancreas [2], and to suppress digestive secretions [3]. SST exists primarily in two functional forms, a canonical 14-amino acid peptide (SST14) and an N-terminally extended 28-amino acid version of this peptide (SST28) [4]. Both versions of the peptide form cyclic structures, due to the presence of a highly conserved disulfide bridge, and are derived from the proteolytic cleavage of the 116-amino acid preprosomatostatin (PPSST), which is coded on Chromosome 3 in humans [4]. Less prominent cleavage products of PPSST exist, including a peptide encompassing residues 31–43 of unknown function, named neuronostatin [5]. SST is assumed to exert its influence primarily through interactions with five cognate G protein-coupled receptors (GPCRs), known as somatostatin receptors (SSTR1 to SSTR5), which are expressed widely throughout the body [6, 7]. Both SST14 and SST28, as well as the closely related paralog cortistatin (CST), activate these receptors through a shared four-amino acid binding epitope comprised of the single-letter amino acid sequence FWKT [8], albeit with different potencies [6].

Given the breadth of SST’s physiological roles, it is no surprise that SST dysfunction is implicated in several human diseases. For decades, SST analogs, including octreotide and lanreotide, have been used to treat neuroendocrine tumors and endocrine disorders, exploiting their ability to mimic the endogenous peptide’s inhibition of excessive hormone release that is often associated with these conditions [9–11]. Because SSTRs are also highly expressed by several other cancer cell types, these receptors increasingly serve as therapeutic cancer targets and biomarkers [12–14]. Several lines of evidence have also linked SST to Alzheimer’s disease (AD) [15–17]. One of the earlier biochemical findings in the AD research field documented reduced levels of SST in postmortem brains of individuals afflicted with the disease [18]. Since then, the key findings of this seminal study have been confirmed by several independent investigators [19–21]. Somatostatinergic neurons moved into the epicenter of AD research a few years later, when two high-profile studies reported that the post-mortem analyses of AD-afflicted brains revealed amyloid β (Aβ) plaques to co-localize with these specialized neurons and neurofibrillary tangles to be predominantly observed in them [22, 23]. Whereas the latter data may suggest a negative influence of somatostatin on AD, the ability of somatostatin to induce the expression of the Aβ-degrading enzyme neprilysin are consistent with a protective role [24]. More recently, AD was genetically linked to the SST gene, when separate reports based on two independent ethnic sample cohorts led the authors to conclude that polymorphisms in this gene may increase the risk of developing AD [25, 26].

We previously used an unbiased mass spectrometry-based affinity-capture approach to identify human brain proteins that bind to the amyloid β (Aβ) peptide, one of the key players in the pathogenesis of AD [27]. This analysis revealed SST as the smallest natural peptide in our dataset that binds selectively to oligomeric Aβ (oAβ). Follow-up validation work revealed that SST interferes with Aβ fibrillization and promotes the formation of distinct SDS-resistant oligomers. In light of the critical role that the amyloidogenic characteristics of Aβ play in AD, it is intriguing to note that SST and several other peptide hormones belong to a group of natural amyloids that are stored in this highly condensed format in secretory granules prior to their cellular release [28]. *In vitro* studies investigating the dynamics and physicochemical aspects of SST aggregation have shown that SST can form laterally associated nanofibrils composed of fixed β-hairpin backbones, similar to other amyloidogenic proteins [29]. The potential significance of these earlier data stems from a plausible scenario, wherein SST might not only interact with oAβ but crosstalk between functional and disease-related amyloidogenic proteins could play a role in the etiology of AD [30].

Based on our Aβ-binding data, we were interested to learn more about SST’s own binding partners and molecular environment. Whereas the principal binding partners of SST, the SSTRs, have been individually isolated and extensively investigated [6, 31–33], to our knowledge, a mass spectrometry-based in-depth analysis of SST-interacting proteins has not been reported. In order to address this gap in the literature, we undertook such a study, using biotinylated SST peptides as baits and human frontal lobe extracts as the biological source for identifying SST-binding candidates. Making use of a previously optimized workflow that includes isobaric tagging for relative and absolute quantitation of proteins (iTRAQ) [34], we report here on a selective interaction between SST and members of the P-type ATPase family. Our data reveal that both SST14 and SST28, but not the similar neuropeptide VIP28, can engage in this interaction. In follow-on work, we characterized the binding epitope and explored how the presence of SST influences the activity of the Na^+^/K^+^-ATPase.

## Results

### Workflow of SST interactome analyses

**Fig 1:**
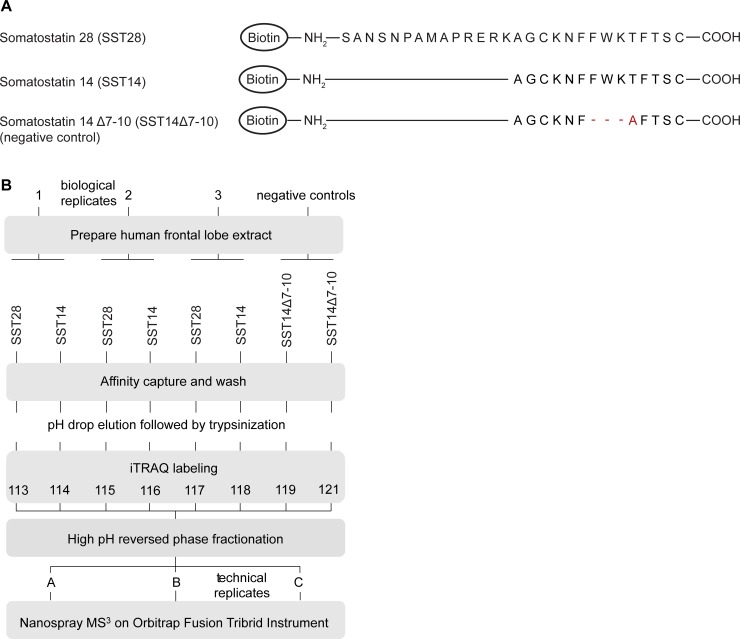
Design of SST interactome experiment. (A) Schematic representation of the bait peptides used in affinity capture experiments. (B) Workflow of SST interactome analysis designed to compare binders of N-terminally biotinylated-SST28 (x) and SST14 (y) to a truncated mutant peptide, SST14Δ7–10 (z).

The purpose of this study was to generate an in-depth inventory of human brain proteins that are capable of binding to SST using an unbiased *in vitro* discovery approach based on affinity capture mass spectrometry. To generate the bait matrices for these analyses, N-terminally biotinylated SST14 or SST28 were pre-bound to commercial streptavidin-conjugated agarose beads (Fig 1A). Human frontal lobe extracts derived from three postmortem brains of individuals that had died of non-dementia causes served as the biological source material. Although SST14 is the predominant form of the peptide, analyses were extended to SST28 out of concern that the small size of SST14 may preclude binding of potential interactors due to steric hindrances caused by the affinity matrix (Fig 1B). Previous investigators had also taken advantage of the 14-amino acid N-terminal extension of SST28 as a natural ‘spacer’ between the biotinyl group and the receptor binding sequence [35, 36] (in this prior work, the SST ligands were, however, added to cultured cells prior to their solubilization in the presence of detergent, an approach that is precluded when dealing with human brain homogenates). As an additional negative control an N-terminally biotinylated SST derivative (hereafter referred to as SST14Δ7–10) was used with the amino acid sequence AGCKNFAFTSC, which lacks the four aforementioned critical residues for SST14 binding to SSTRs (Fig 1A). Sample groups were analyzed side-by-side in triplicate. The zwitterionic detergent 3-((3-cholamidopropyl) dimethylammonio)-1-propanesulfonate (CHAPS) was used to solubilize membrane proteins in human frontal lobe samples, a choice based on previous reports that established the ability of this detergent to extract the SSTRs [35, 37]. Following overnight incubation of the brain extracts with affinity matrices, bound proteins were eluted by rapid acidification, denatured in 9 M urea, reduced, alkylated and trypsinized. To avoid inadvertent variances between samples, which are notoriously observed when mass spectrometry analyses are undertaken consecutively, individual tryptic digests were labeled with distinct iTRAQ labels in an eight-plex format and then combined. The iTRAQ-labeled mixture was purified using microscale reversed phase (RP) chromatography at low pH in parallel with high pH reversed phase fractionation. Mass spectra for peptide sequencing and quantification were obtained over four-hour RP nano-HPLC separations with simultaneous data collection on an Orbitrap Fusion Tribrid mass spectrometer running an MS/MS/MS (MS^3^) analysis method. The relative levels of peptides in the eight samples were determined by comparing the intensity ratios of the corresponding low mass iTRAQ signature ions in MS^3^ fragment spectra.

### The SST interactome

**Fig 2:**
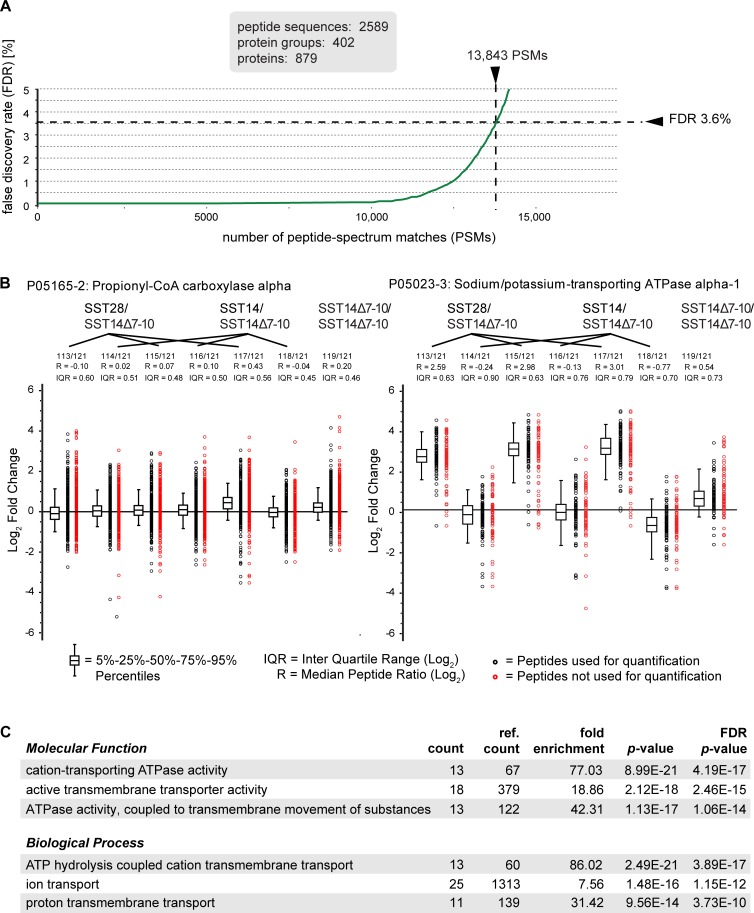
Benchmarks of SST interactome analysis and preliminary data. (A) The number PSMs versus the false discovery rate (FDR) in the interactome dataset. (B) Relative quantitation of propionyl-CoA carboxylase, which displayed similar levels of enrichment across all samples, and Na^+^/K^+^-transporting ATPase subunit alpha-1, which was highly enriched in biotin-SST28 affinity capture eluates. The box plot depicts the enrichment ratios (R) of individual propionyl-CoA carboxylase peptides used for quantitation in log2 space, in addition to the median peptide ratio and Inter Quartile Range (IQR). A subset of peptides (red circles) was eliminated from the quantitation due to redundancy or failure to pass stringency thresholds. Relative protein levels are depicted as ratios, with the ion intensities corresponding to the heaviest isobaric labels acting as the reference. (C) ‘Cellular Component’ and ‘Molecular Function’ Gene Ontology analyses of the shortlisted proteins that displayed the highest enrichment in biotin-SST28 affinity capture eluates.

The combined SST interactome analyses from three biological replicates yielded peptide sequence assignments to 13,843 mass spectra, which matched to 402 protein groups, comprising a total of 879 unique proteins, with a false discovery rate (FDR) of 3.6% (Fig 2A). Stringent filtering on the basis of the quality of quantitation data, i.e., requiring proteins to be quantified by a minimum of three iTRAQ reporter ion profiles and to exhibit low (< 30%) deviation in their relative levels between biological replicates, reduced the list of SST candidate interactors to 88 proteins (please see Table 1 for a truncated list of proteins identified, sorted according to their average enrichment in the three biotin-SST28 affinity capture eluates, and S1 Table for a full list of candidate interactors, sorted alphabetically). Propionyl-CoA carboxylase, an endogenously biotinylated enzyme ubiquitously expressed in eukaryotic cells [38] bound the streptavidin affinity capture matrix independently of the SST bait peptides, serving as an internal control (Fig 2B; S1 Table). Each of the two propionyl-CoA carboxylase subunits was identified from more than 1,850 tandem mass spectra, giving rise to the highest protein sequence coverage in the data set. Consistent levels of propionyl-CoA carboxylase subunits A (CV = 0.13) and B (CV = 0.15) across all eight eluates indicated that the lysates contained similar levels of brain proteins and that capture conditions were comparable across all samples.

**Table 1:** Top-listed 30 interactors of biotin-SST28. Proteins are listed according to their average enrichment in the three biotin-SST28 eluates.

Accession	Description^1^	Coverage	Total Peptides	PSMs	SST28/ SST14Δ7–10 (113/121)	SST28/ SST14Δ7–10 (115/121)	SST28/ SST14Δ7–10 (117/121)	Counts	AAs
P05023-3	Sodium/potassium-transporting ATPase subunit alpha-1	36.39%	31	337	6.035	7.896	8.037	98	992
M0R116	Sodium/potassium-transporting ATPase subunit alpha-3	31.23%	24	275	5.937	8.073	7.941	83	983
B1AKY9	Sodium/potassium-transporting ATPase subunit alpha-2	33.99%	28	236	5.961	8.187	7.331	63	1009
B4DWR3	Prefoldin subunit 3	21.35%	3	9	5.253	6.896	7.413	6	192
Q16623	Syntaxin-1A	28.82%	5	13	3.580	9.347	5.994	3	288
P11216	Glycogen phosphorylase, brain form	33.10%	24	132	6.234	5.328	7.310	34	843
P11217-2	Glycogen phosphorylase, muscle form	29.58%	17	45	7.345	4.916	6.313	11	754
P61266-2	Syntaxin-1B	30.69%	7	34	3.800	7.007	7.564	19	277
P60880	Synaptosomal-associated protein 25	48.06%	9	80	4.835	6.869	4.920	20	206
Q7L0J3-2	Synaptic vesicle glycoprotein 2A	5.72%	5	8	4.573	5.145	5.385	3	682
P20020-5	Plasma membrane calcium-transporting ATPase 1	20.50%	16	77	4.284	5.373	5.088	18	1171
O75746-2	Calcium-binding mitochondrial carrier protein Aralar1	29.07%	12	29	3.465	5.66	5.246	5	571
P43004-3	Excitatory amino acid transporter 2	19.54%	8	33	4.041	5.362	4.310	13	563
J3KQA0	Synaptotagmin I	17.66%	7	20	1.629	8.633	3.173	7	419
P52209-2	6-phosphogluconate dehydrogenase, decarboxylating	22.55%	10	25	3.863	4.986	4.508	8	470
Q08209	Serine/threonine-protein phosphatase 2B subunit alpha	21.88%	10	65	3.429	3.793	4.320	18	521
F6U1T9	Calcineurin subunit B type 1	34.38%	4	7	1.963	5.384	3.544	3	160
P09471	Guanine nucleotide-binding protein G(o) subunit alpha	33.90%	10	93	3.076	4.298	3.318	34	354
P38606-2	V-type proton ATPase catalytic subunit A	24.32%	10	48	3.769	2.434	4.157	9	584
Q01814	Plasma membrane calcium-transporting ATPase 2	19.71%	17	44	2.835	3.348	4.074	7	1243
Q96F85	CB1 cannabinoid receptor-interacting protein 1	17.68%	3	5	2.792	3.414	3.803	4	164
P12235	ADP/ATP translocase 1	55.03%	14	35	1.949	2.421	5.392	9	298
P05091	Aldehyde dehydrogenase, mitochondrial	26.89%	15	63	2.422	3.618	3.028	19	517
Q00325	Phosphate carrier protein, mitochondrial	14.36%	4	25	1.863	2.490	3.885	11	362
P05141	ADP/ATP translocase 2	42.95%	14	44	1.618	2.380	4.217	18	298
P12236	ADP/ATP translocase 3	46.98%	14	48	1.345	2.373	4.402	21	298
O00154	Cytosolic acyl coenzyme A thioester hydrolase	35.00%	10	32	2.740	2.238	3.054	13	380
E9PE24	Visinin-like protein 1 (Fragment)	37.50%	4	11	2.503	2.246	2.868	7	104
Q16698	2,4-dienoyl-CoA reductase, mitochondrial	27.76%	7	18	2.586	2.266	2.368	13	335
X6RFL8	Ras-related protein Rab-14 (Fragment)	32.60%	5	16	2.439	2.750	1.852	6	181

^1^This list was derived from S1 Table by applying a filter that excluded proteins displaying greater than 50% variation in enrichment between the two SST14Δ7–10 control replicates. The list has been truncated to fit on the page.

As a first step in the characterization of candidate SST-binders, the 50 proteins most selectively bound to SST28 capture matrices were subjected to a Gene Ontology (GO) analysis, which revealed enrichment of proteins involved in ion transport. The molecular functions ‘cation-transporting ATPase activity,’ ‘active transmembrane transporter activity,’ and ‘ATPase activity, coupled to transmembrane movement of substances’ were overrepresented (Fig 2C). Similarly, ‘ATP hydrolysis coupled cation transmembrane transport,’ ‘ion transport,’ and ‘proton transmembrane transport’ were top-listed, highly significant biological processes (Fig 2C). Consistent with this result, the most selectively enriched SST28 candidate binders included Na^+^/K^+^-transporting ATPase, excitatory amino acid transporter, V-type proton ATPase, and ADP/ATP translocase (Table 1). In addition to transporters, SNARE protein family members syntaxin, synaptosomal-associated protein 25 (SNAP25), and synaptotagmin were identified as candidate SST28 binders. These proteins, along with other members of the SNARE complex, are known to be involved in the fusion of synaptic vesicles with their target membranes. Other candidate SST28 interactors included the catalytic (serine/threonine-protein phosphatase 2B) and regulatory (calcineurin subunit B) subunits of the calcium-dependent protein phosphatase calcineurin. SSTRs themselves, however, were not observed (please see Discussion for interpretation). Taken together, these results were unexpected and pointed toward an unappreciated interaction of SST with members of the family of P-type ATPases and SNARE protein complexes.

### SST28 binds to multiple members of the P-type ATPase superfamily

**Fig 3:**
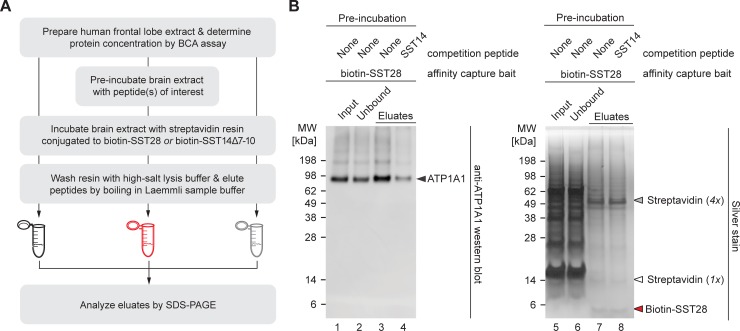
Identification of ATP1A1 as a candidate interactor of SST28/14. (A) Workflow of competitive binding experiments undertaken to validate the SST-Na^+^/K^+^-transporting ATPase subunit alpha-1 interaction. (B) SDS-PAGE analysis of SST28 affinity capture eluates visualized by immunoblot and silver stain. Immunoblot against ATP1A1 reveals that binding of ATP1A1 to biotin-SST28 can be blocked by pre-incubation of the brain lysate with free SST14 (50 μM; lane 4). The prominent bands in the eluates corresponding to ATP1A1 are absent when the same samples are analyzed by silver stain (lanes 7, 8). Note the presence of intense bands in the input and unbound fractions (lanes 5, 6) that are absent from SST28 affinity capture eluates, indicating that SST is not inclined to interact with other proteins simply based on their abundance. The bands at approximately 50, 14, and 4 kDa in the eluate samples likely correspond to streptavidin subunits and biotin-SST28 peptides that leached off the affinity capture resin during the elution step, given their absence from the input and unbound fractions.

Among the P-type ATPases that showed the strongest levels of SST-dependent enrichment were subunits of the Na^+^/K^+^-ATPase (alpha-1, alpha-2, alpha-3, beta-1) and isoforms 1 and 2 of the plasma membrane Ca^2+^-transporting ATPase. In fact, the alpha-1 subunit of the Na^+^/K^+^-ATPase (ATP1A1) displayed the highest level of SST co-enrichment amongst all SST candidate interactors (Table 1), ranging from 6 to 8 times the levels observed in SST14Δ7–10 negative control eluates (Fig 2B). These proteins were enriched in biotin-SST28 affinity capture eluates but not in biotin-SST14 eluates. To determine if steric hindrance caused by tethering SST14 to the affinity matrix precluded binding of ATP1A1, affinity capture experiments were conducted in which the ability of free SST14 to compete for binding to the biotin-SST28 bait matrix was assessed (Fig 3A). The subsequent immunoblot assessment of assay fractions validated that ATP1A1 is, indeed, captured by biotin-SST28 (Fig 3B). Moreover, pre-incubation of the brain lysate with free SST14 diminished the capture of ATP1A1 by biotin-SST28, consistent with the interpretation that SST14 can also bind to the Na^+^/K^+^-ATPase when it is not sterically restrained. Considering the tendency of abundant proteins to contaminate affinity purified preparations, the concern arose whether ATPase capture was indeed SST specific or merely reflected the high relative protein levels of these P-type pumps in the brain. Silver staining of the affinity capture eluates revealed that washing of the capture matrix had removed many of the most abundant proteins (Fig 3B). In fact, only a small subset of proteins visible in the input samples were retained by the SST28 matrix. Collectively, these findings established that both SST28 and SST14 interact selectively with Na^+^/K^+^-ATPases.

### Characterization of SST binding to the Na^+^/K^+^-ATPase

**Fig 4:**
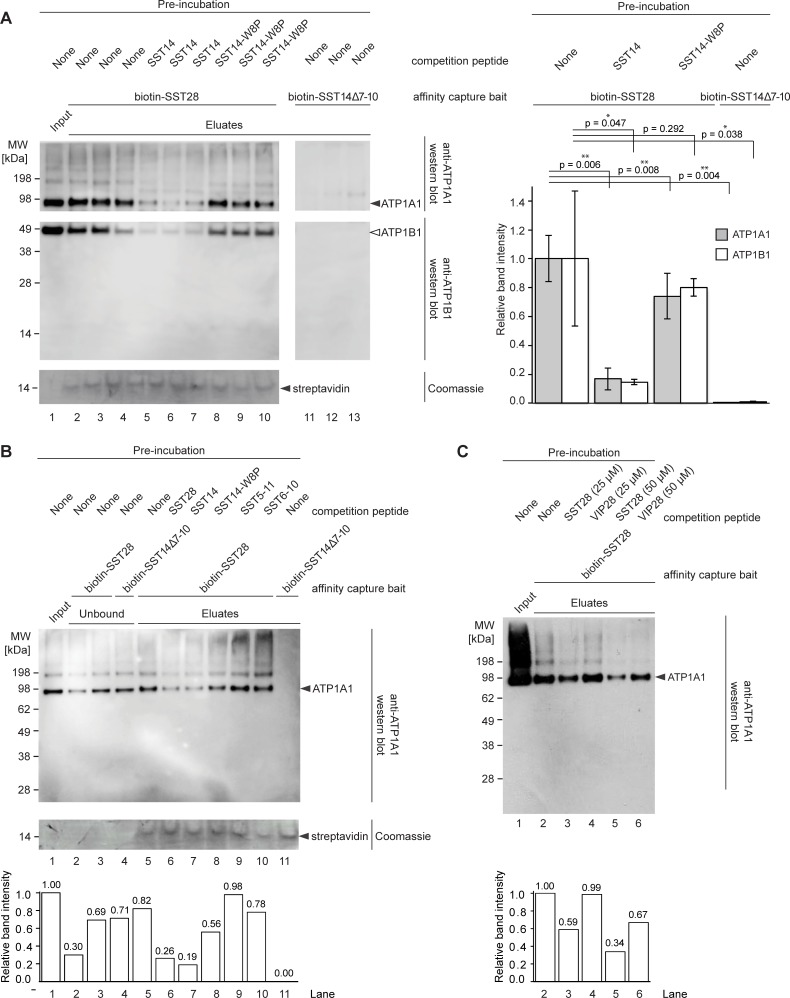
Validation of SST binding to Na^+^/K^+^-ATPase alpha and beta subunits. (A) Immunoblot analysis of competitive binding experiment with SST14 and the mutant peptide SST14-W8P (left) and quantification of western blot data (right). Capture of ATP1A1 and ATP1B1 by biotin-SST28 can be blocked by pre-incubation of the brain lysate with free SST14 (50 μM; lanes 5–7), but not with a mutant SST14 with an amino acid substitution (W8P) in the receptor-binding site (50 μM; lanes 8–10). Note that the negative control bait peptide, biotin-SST14Δ7–10, failed to capture any detectable ATP1A1 (lanes 11–13). Coomassie staining of the same blot confirms that an equal amount of protein was loaded in each well, using the streptavidin subunits released from the affinity matrix as a loading control (lower panel). National Institutes of Health (NIH) Image J densitometry analyses of the anti-ATP1A1- and anti-ATP1B1-reactive bands appearing near the 98 kDa and 49 kDa molecular weight markers are shown in the panel to the right (B) Capture of ATP1A1 by biotin-SST28 can be blocked by pre-incubation of the brain lysate with free SST28 (50 μM; lane 6) or SST14 (50 μM; lane 7), but not with SST14-W8P (50 μM; lane 8). Truncated versions of SST14 also fail to block capture of ATP1A1 (50 μM; lanes 9, 10), despite containing the receptor binding sequence (FWKT). Coomassie staining confirms that protein loading was consistent across the gel (middle panel). NIH ImageJ densitometry of the anti-ATP1A1-reactive band appearing is shown in the lower panel (C) Capture of ATP1A1 by biotin SST-28 can be blocked by pre-incubation of the brain lysate with free SST28 (25 μM or 50 μM; lanes 3, 5) but not the similar neuropeptide, VIP (25 μM or 50 μM; lanes 4,6) as shown by western blot (upper panel) and densitometry of the anti-ATP1A1-reactive band (lower panel).

To begin to delineate the epitope within SST required for its Na^+^/K^+^-ATPase affinity, we next performed a series of competitive binding experiments (Fig 3A) using various SST-derived peptides. Specifically, again using biotin-SST28 or biotin-SST14Δ7–10 as baits, human brain lysates were pre-incubated with either SST14 (50 μM) or a mutated version of the peptide, SST14-W8P (50 μM), which contains a tryptophan to proline substitution in the previously determined SSTR binding epitope. Immunoblot analyses validated binding of SST28 to ATP1B1, the beta-1 subunit of the Na^+^/K^+^-ATPase, in addition to ATP1A1. As expected, both interactions could be blocked by pre-incubation of the brain lysate with SST14 (Fig 4A). Additionally, these experiments revealed that the single amino acid substitution in the receptor binding site of SST prevented its binding to these proteins. This inference was based on the observation that pre-incubation with SST14-W8P did not impair the interaction of ATP1A1 or ATP1B1 with biotinylated-SST28 (Fig 4A). Analogous competition experiments in which N- and C-terminal truncated SST14 derivatives SST5-11 or SST6-10 were used as blocking peptides indicated that these peptides, although containing the critical tryptophan-8 residue, did not compete with SST28 for binding to Na^+^/K^+^-ATPase (Fig 4B). In other words, the core SSTR binding sequence FWKT within SST seemed to be essential but insufficient for binding the Na^+^/K^+^-ATPase.

To further assess the specificity of the interaction between SST and P-type ATPases, the binding competition analyses were extended to vasoactive intestinal peptide (VIP), another 28-amino acid neuropeptide whose tissue expression overlaps with SST, with both peptides being present in the gastrointestinal tract and hypothalamus [39]. Pre-incubation of human brain extract with either SST28 or VIP revealed that SST28, but not VIP, was able to block the binding of biotin-SST28 bait to ATP1A1 (Fig 4C), further corroborating the conclusion that there is specificity to the interaction between SST and the Na^+^/K^+^-ATPase.

We explored the possibility that SSTRs and ATP1A1 interact with SST via similar binding domains. To this end, we compared the sequences of all five human SSTRs and all four human alpha-subunits of Na^+^/K^+^-ATPases in the Uniprot database. This analysis revealed that SSTRs have low sequence identity with alpha-subunits. For example, the pairwise comparison of all SSTRs with ATP1A1 revealed sequence identities that ranged between 9.9% (SSTR4) and 12.9% (SSTR3) (not shown). Although the precise binding mode of SST to SSTRs is not known, due to the absence of a high-resolution structure, this result is in line with data which suggest that SST binds within SSTRs via a conformational discontinuous epitope [40].

### SST inhibits ^86^Rb uptake by the Na^+^/K^+^-ATPase

**Fig 5:**
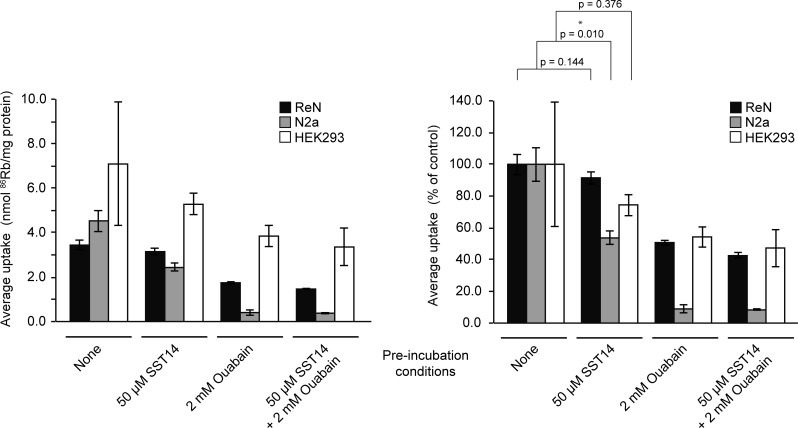
SST14 regulates ^86^Rb^+^ uptake by the Na^+^/K^+^-ATPase in ReN, N2a and HEK293 cells. The addition of SST14 (50 μM) to ReN, N2a and HEK293 cells reduces the uptake of ^86^Rb^+^ to 91.5%, 53.6% and 74.4%, respectively, compared to controls. Following treatment with ouabain (2 mM) the uptake of ^86^Rb^+^ is reduced to 50.7% in ReN cells, 8.9% in N2a cells and 54.3% in HEK293 cells, respectively. Note that the addition of SST14 to ouabain treated cells does not further reduce the uptake of ^86^Rb^+^ compared to cells treated with ouabain alone.

In order to address whether the interaction between SST and the Na^+^/K^+^-ATPase has an effect on the activity of the pump, we undertook an *in vitro* ^86^Rb^+^ uptake assay in human neural progenitor (ReNcell VM) and mouse neuroblastoma (N2a) cell lines, which was adapted from previously established protocols [41]. We also included wild-type human embryonic kidney (HEK293) cells in these analyses, because these cells express negligible levels of SSTRs, yet are known to endogenously express the alpha-1 subunit of the Na^+^/K^+^-ATPase and to exhibit robust pump-dependent ion uptake [42]. Experiments were performed in the absence and presence of ouabain, a potent inhibitor of the Na^+^/K^+^-ATPase, to discern the Na^+^/K^+^-ATPase-dependent and -independent uptake of ^86^Rb^+^, a radionuclide which pharmacologically mimics K^+^. In the presence of SST14 (50 μM), total ^86^Rb^+^ uptake was reduced to 91.5%, 53.6% and 74.4% in ReN, N2a and HEK293 cells, respectively, compared to controls (Fig 5). In contrast, total ^86^Rb^+^ uptake was reduced to 50.7% in ReN cells, 8.9% in N2a cells and 54.3% in HEK293 cells following treatment with ouabain (2 mM). The ouabain-mediated reduction of ^86^Rb^+^ uptake was not potentiated by the treatment of cells with both ouabain (2 mM) and SST14 (50 μM), consistent with the interpretation that the inhibitory effect of SST14 on ^86^Rb^+^ uptake is mediated by the Na^+^/K^+^-ATPase. These findings indicate that SST14 reduced the activity of the Na^+^/K^+^-ATPase in a cell type-specific manner, with N2a cells being particularly susceptible to SST-mediated inhibition, HEK293 cells exhibiting highest levels of ^86^Rb^+^/mg internalization but being only partially susceptible to SST-mediated inhibition, and ReN cells displaying resistance to SST-mediated Na^+^/K^+^-ATPase inhibition.

## Discussion

The goal of the current study was to generate an inventory of human brain proteins that bind to SST. Biotin-SST affinity capture robustly enriched 88 proteins each with at least 3 confident peptide-spectrum matches (PSMs). Among the top SST candidate interactors were multiple members of the P-type ATPase superfamily. Follow-up experiments, which centered on the Na^+^/K^+^-ATPase, validated that both SST28 and SST14 can bind to this pump. We then observed that binding exhibits selectivity with regards to both the SST bait peptide when compared to similar peptides, and to the P-type ATPase prey when compared to other abundant brain proteins. Moreover, we identified a tryptophan residue within SST that appears to be critical for binding to the Na^+^/K^+^-ATPase. Interestingly, this tryptophan is embedded within the core FWKT sequence motif known to also mediate binding of SST to its cognate receptors. Finally, we observed that SST has a cell type-specific inhibitory effect on the activity of the Na^+^/K^+^-ATPase.

Our study took advantage of recent improvements to mass spectrometry instrumentation and advanced workflows that incorporated isobaric labeling for relative quantitation. Since we sought to identify any protein that might interact with SST in the brain, a rather generic affinity capture approach was applied. In hindsight we realized that this strategy disfavored the purification of canonical SSTRs which, as members of the GPCR protein family, become unstable and lose their ligand-binding ability when removed from their native environment in a manner that disrupts their physiological interaction with G-proteins [43–45], necessitating cross-linking and modified affinity-capture protocols to stabilize the interaction of SST with its canonical receptors [36, 45, 46]. This limitation may also extend to other GPCRs, including opioid and dopamine receptors, which had been implicated in SST-dependent phenotypes by others [47, 48], possibly on account of an inherent propensity of many GPCRs to operate as homo- or heterodimers [49].

A PubMed query that combined the search terms ‘somatostatin’ and ‘mass spectrometry’, although producing more than 150 hits, revealed no prior study that pursued the objective of identifying SST interacting proteins. Close examination of the pertinent literature suggests that this striking omission may at least in part reflect the fact that the discovery of canonical SSTRs in 1992 predated technological developments underlying modern protein mass spectrometry. Consequently, the first two canonical SSTRs (SSTR1 and 2) were identified through a genomic hybridization strategy, which specifically targeted GPCRs of pancreatic islet cells because earlier research had suggested SSTRs to be members of this receptor family [50]. The subsequent discoveries of SSTR3, 4 and 5 followed in short order, and were based on conceptually analogous genomic hybridization screens [51–53]. Thus, whereas until just before that time, the focus in the pertinent literature had been on the purification of SST receptors by biochemical means, this line of research was largely abandoned after 1992. When SSTR1 and SSTR2 were expressed in CHO cells, binding experiments with radiolabeled SST probes revealed that approximately 90% of binding depended on the presence of the heterologous SSTRs [31]. These and similar results obtained with the other SSTR paralogs may have limited the motivation to look any further, although they did not rule out the existence of additional physiological SST interactors. Consistent with the view that the known SSTRs may not account for all binders of SST, leading up to 1992, the molecular masses of candidate SST interacting proteins were reported to range from 27 to 228 kDa [54], and the masses of canonical SSTRs of approximately 40 kDa would have been difficult to reconcile with data of several investigators in the field.

The most striking finding from the current study was a selective interaction between SST and members of the P-type ATPase superfamily. In addition to P-type pumps, our interactome study revealed several other novel candidate SST interactors. Some of these had been indirectly linked to SST. For example, both subunits of the heterodimeric protein calcineurin, i.e., the catalytic serine/threonine-protein phosphatase 2B (PPP3CA) and its regulatory subunit (PPP3R1), emerged in this work as candidate SST binders. Calcineurin had previously been proposed to operate as a downstream effector of SSTRs [55]. Similar levels of enrichment were observed for members of the synaptic vesicle fusion complex, including syntaxin-1A/B, SNAP25, synaptotagmin, and synaptic vesicle glycoprotein 2A.

Focusing on the Na^+^/K^+^-ATPase for validation studies, we demonstrated that a moiety overlapping with the SSTR binding sequence FWKT is required by both SST28 and SST14 to interact with this pump. Even more intriguingly, we previously reported that this region of SST is also critical for its binding to oligomeric Aβ [27, 30], an observation that triggered our interest in other SST interactors in the first place. Indicative of some specificity of this interaction, other abundant proteins (like tubulin and myelin basic protein) were not captured by the matrix, the mere replacement of a single tryptophan residue within SST made it non-competitive for binding to the pump, and the neuropeptide VIP, of similar mass (SST28 = 3.2kDa, VIP = 3.3kDa) and physicochemical characteristics (equal length, SST28 pI = 9.85, VIP pI = 9.82, SST28 GRAVY = -0.732, VIP GRAVY = -0.639) as SST, failed to block the capture of ATP1A1 by biotin-SST28. Although this is the first report of a direct binding between SST and P-type ATPases, prior to the discovery of SSTRs multiple authors identified proteins that bound selectively to SST analogs with molecular weights suspiciously similar to the alpha subunit of the Na^+^/K^+^-ATPase (~100 kDa) [46, 56, 57]. Furthermore, SST has been observed to modulate plasma membrane conductance in various cell types [58–62]. The primary method by which this was proposed to occur was through binding to GPCRs, which can hyperpolarize the membrane by opening K^+^ channels and by lowering intracellular Ca^2+^ levels [63]. For example, SST was reported to activate inwardly rectifying K^+^ channels and inhibit voltage gated Ca^2+^ entry through the action of different G-proteins [59–62, 64–66] leading to hyperpolarization of the cell membrane [67]. It will be of interest to explore if SST additionally affects membrane conductance by directly binding to and modulating the activity of P-type ATPases and whether such interactions could have an impact on synaptic plasticity, learning, and memory. We have shown here that SST exposure leads to a partial inhibition of the Na^+^/K^+^-transporting ATPase in certain cell types. Similarly, SST may bind to and modulate the activity of the Ca^2+^-transporting ATPase, another P-type ATPase we co-isolated, which would be expected to affect intracellular Ca^2+^ levels.

SST may also indirectly influence membrane conductance through a different mechanism: Binding of SST to its cognate receptors is best understood to activate G_αi_, a subunit of heterotrimeric G proteins. G_αi_ inhibits adenylate cyclase, thereby reducing cAMP production, which leads in several cell models to a decrease in the levels and activity of the Na^+^/K^+^-ATPase [68, 69]. If direct binding of SST to the Na^+^/K^+^-ATPase inhibits the pump, it could constitute an elegant signal amplification mechanism working synergistically with SSTR activation to decrease the levels and activity of the Na^+^/K^+^-ATPase.

A related consideration is the role of SST in controlling vascular tone. Na^+^/K^+^-ATPases are a primary pharmacological target for the treatment of hypertension using cardiotonic glycosides [70]. Multiple lines of evidence suggest that SST also has vasoactive properties, and generally acts as a hypertensive agent. In fact, for many years, somatostatin analogs have been used in the treatment of portal hypertension by inducing vasoconstriction of the splanchnic vasculature [71–73]. The identification of a selective interaction between SST and the Na^+^/K^+^-ATPase might be pertinent in this context, with potential implications for administering SST analogs in the clinic.

## Materials and methods

### Peptides

Synthetic human peptides, including SST14 (AS-24277), SST28 (AS-22902), and VIP (AS-22873) were purchased from Anaspec, Inc. (Fremont, CA, USA). Other peptides, including biotinylated SST peptides (biotin-SST14, biotin-SST28, and biotin-SST14Δ7–10) and the truncated/mutant SST14 peptides (SST14-W8P, SST5-11, SST6-10) were custom synthesized by LifeTein LLC (Hillsborough, NJ, USA).

### Antibodies

Immunoblot analyses made use of the anti-sodium/potassium alpha 1 ATPase antibody (ab7671; Abcam Inc., Toronto, ON, Canada) and the anti-sodium/potassium beta 1 ATPase antibody (GTX113390; GeneTex Inc., Irvine, CA, USA). Anti-mouse (170–6516) and anti-rabbit (170–6515) horseradish peroxidase-conjugated secondary antibodies were sourced from Bio-Rad Laboratories, Inc., Hercules, CA, USA.

### Western blot, Coomassie, and silver staining

For SDS-PAGE analyses, samples were mixed with Bolt LDS sample buffer (B0007; Thermo Fisher Scientific, Burlington, ON, Canada) containing 2.5% 2-mercaptoethanol and boiled for 10 minutes at 60°C before loading. The samples were separated on Bolt 10% Bis-Tris Plus gels (NW00102BOX; Thermo Fisher Scientific, Burlington, ON, Canada) in MES SDS Running Buffer (NP0002; Thermo Fisher Scientific, Burlington, ON, Canada) for 1 to 1.5 hr at 120 V. For immunoblot analyses, peptides were transferred to polyvinylidene difluoride (PVDF) membranes at 50 V in Tris-Glycine buffer containing 10–20% methanol for 2 hr. Membrane blocking steps were done in standard Tris-buffered saline with 0.1% Tween 20 (TBST) containing 5% fat-free milk and membranes were incubated overnight with the appropriate primary antibodies for antigen binding. Following three washes with TBST, membranes were incubated for 1 hr with 1: 2000 diluted anti-mouse or anti-rabbit horseradish peroxidase-conjugated secondary antibodies. The band signals were visualized using enhanced chemiluminescence reagents (4500875; GE Health Care Canada, Inc., Mississauga, ON, Canada) and X-ray films or a LI-COR Odyssey Fc digital imaging system (LI-COR Biosciences, NE, USA). Where indicated, Coomassie and/or silver staining were performed to visualize all proteins present in the sample.

### Affinity capture of SST14- and SST28-binding proteins from human frontal lobe extracts

Streptavidin UltraLink Resin beads (53114; Thermo Fisher Scientific, Burlington ON, Canada) were chosen as the affinity capture matrix in this study. The biotinylated SST14, SST28, and SST14Δ7–10 peptides were captured on the resin by 2 hr incubation in PBS at room temperature while undergoing continuous agitation on a slow-moving turning wheel. The biological source material for the interactome analysis consisted of human frontal lobe tissue samples from individuals (3 males) who died of non-dementia related causes at ages of 74, 76 and 82 years. The brains were obtained from the tissue biobank at the Tanz Centre for Research in Neurodegenerative Diseases, where they had been stored in -80°C freezers. 150 mg pieces of each brain were used per experimental condition and were homogenized in lysis buffer containing cOmplete Protease Inhibitor Cocktail (11836170001; Roche, Mississauga, ON, Canada) and PhosStop phosphatase inhibitor tablets (04906837001; Roche, Mississauga, ON, Canada) and solubilized using 0.6% CHAPS detergent (C3023; Sigma-Aldrich, Oakville, ON, Canada). For follow-up validation experiments, 30 mg pieces of brain sample were used. Following centrifugation at 21,000 g for 1 hr to remove insoluble material, the protein concentration was normalized across all samples. The brain lysate was subsequently added to the pre-saturated affinity capture beads (20 μL per biological replicate) and incubated overnight at 4°C. The affinity capture beads were then extensively washed during three wash steps with a total of 60 mL of lysis buffer containing 150–500 mM NaCl. Prior to elution, the affinity matrix was subjected to a pre-elution wash with 15 mL of 10 mM HEPES that served to reduce detergent and salt levels. Captured proteins were eluted either by rapid acidification in a solution containing 0.2% trifluoroacetic acid and 20% acetonitrile in deionized water (pH 1.9) (for mass spectrometry) or by boiling for 10 min at 60°C in Bolt LDS sample buffer (B0007; Thermo Fisher Scientific) containing 2.5% 2-mercaptoethanol (for western blot analyses).

### Cell culture

Human neural progenitor (ReNcell VM) cells (SCC008; Millipore Sigma, Etobicoke, ON, Canada) were cultured according to the manufacturer’s instructions. Mouse neuroblastoma Neuro-2a (N2a) cells (CCL-131; American Type Culture Collection, Manassas, VA, USA) and human embryonic kidney (HEK293) cells (CRL-1573; American Type Culture Collection, Manassas, VA, USA) were grown in Dulbecco’s Modified Eagle Medium (DMEM) supplemented with 10% v/v heat inactivated fetal bovine serum (FBS) (catalog number 12484028; Invitrogen Canada, Burlington, ON, Canada), 1% GlutaMAX (catalog number 35050061; Invitrogen Canada), and antibiotics in a 24 well format.

### ^86^Rb^+^ uptake assay

The ^86^Rb^+^ uptake assay procedure was based on previously established protocols [41]. More specifically, three hr before each experiment, the cell culture medium was replaced with DMEM containing 0.2% FBS. Following serum deprivation, the cells were treated with ouabain (2 mM) (O3125; Sigma-Aldrich, Oakville, ON, Canada) and/or SST14 (50 μM) or with vehicle (control). After incubation for 15 min at room temperature, 2 μCi ^86^RbCl in water (NEZ072; PerkinElmer, Woodbridge, ON, Canada) was added to each well and the cells were incubated for another 10 min at 37°C. The supernatants were then removed, and cells were washed four times with 1 mL ice-cold wash buffer (100 mM MgCl_2_, 10 mM HEPES, pH 7.4) before lysis in 500 μL buffer containing 1% NP40, 150 mM Tris (pH 8.3), and 150 mM NaCl. 250 μL aliquots of the cell lysates were transferred to vials containing 10 mL Ultima Gold liquid scintillation cocktail (6013326; PerkinElmer, Woodbridge, ON, Canada) and assayed using a liquid scintillation counter (LS6500; Beckman Coulter; Mississauga, ON, Canada). Additional 10 μL aliquots were used for protein concentration determination using BCA reagents (23228 and 1859078; Thermo Fisher Scientific, Burlington, ON, Canada).

### Sample preparation for interactome analysis

Processing of the affinity-capture eluates followed previously described protocols [27, 74]. First, the organic solvent was removed from the samples using a centrifugal evaporator. The acidity of the sample was reduced by the addition and continuous evaporation of an additional three volumes of water. Two volumes of 9 M urea were then added per volume of sample, and protein denaturation was allowed to take place over 10 minutes at room temperature. The pH was further adjusted by the addition of 100 mM HEPES (pH 8.0). Following reduction for 30 minutes at 60°C in the presence of 5 mM tris (2-carboxyethyl) phosphine (TCEP), proteins were alkylated for 1 hr at room temperature in 10 mM 4-vinylpyiridine (4-VP). Protein mixtures were diluted with 500 mM tetraethylammonium bicarbonate (TEAB; pH 8.0) to a total volume of 100 microliters to ensure that urea concentrations were not in excess of 1.5 M. Digestion of samples with side-chain-modified porcine trypsin (90057; Thermo Fisher Scientific, Burlington, ON, Canada) proceeded overnight at 37°C. Primary amines were covalently modified with isobaric tagging for relative and absolute quantitation (iTRAQ) reagents (4381663; SCIEX, Concord, ON, Canada) by following the manufacturer’s instructions. The labeled digests were then pooled into a master mixture and purified with C18 (A5700310; Agilent Technologies, Inc., Mississauga, ON, Canada) or a high pH reversed phase fractionation kit (84868, Thermo Fisher Scientific, Burlington, ON, Canada), again following the manufacturer’s instructions. Finally, upon reconstitution in 0.1% formic acid, peptides were analyzed by tandem mass spectrometry on an Orbitrap Fusion Tribrid instrument using previously described parameters [27].

### Post-acquisition data analyses

The post-acquisition data analysis of interactome data sets was conducted against the Uniprot canonical and isoform human database (October 29, 2017 version, downloaded January 3, 2018), which was queried with Mascot (Version 2.4.1; Matrix Science Ltd, London, UK) and Sequest HT search engines within Proteome Discoverer software (Version 1.4; Thermo Fisher Scientific, Burlington, ON, Canada). Protein sequence and quantification data complimentary to that produced on Proteome Discoverer was created using PEAKS Studio software (Version 8.5; Bioinformatics Solutions Inc., Waterloo, ON, Canada). A maximum of two missed tryptic cleavages and naturally occurring variable phosphorylations of serines, threonines and tyrosines were allowed. Other variable modifications considered were oxidation of methionine, tryptophan and histidine as well as deamidation of glutamine or asparagine. Mass spectrometry data sets have been deposited to the ProteomeXchange Consortium [75] via the PRIDE partner repository [76] with the project name ‘Somatostatin-interacting human brain proteins’ and the dataset identifier PXD010885 (http://proteomecentral.proteomexchange.org/cgi/GetDataset).

### Statistical analyses

In the LC-MS/MS data interpretation by Proteome Discoverer, which was undertaken as described before [77], peptide sequencing quality was maintained at cut off scores providing an FDR of 0.05, estimated by the Percolator algorithm [78], with PSMs scoring below these cut offs excluded from quantification. Percolator analysis was conducted using a maximum delta Cn (PSM rank for each sequenced mass spectrum) of 0.05 and validation based on q-value. Protein sequencing with the PEAKS algorithm was performed at peptide and protein cut off scores of 0.0316 and 0.01 respectively.

Gene ontology analyses were conducted using the PANTHER Overrepresentation Test via the GO consortium online tool (http://geneontology.org/) under default settings.

For comparisons between two groups, statistical analyses were based on the paired Student’s t-test, where a *p*-value of less than 0.05 was considered significant. In instances when the experimental design was predictive of a one-directional change these tests were one-tailed. Standard errors of the mean are represented as error bars in the figures. Western blot and *in vitro* experiments were performed in triplicate where statistical analyses were implemented. For better visualization of the western blot quantifications, data were normalized to the control condition (no peptide pre-incubation) and relative standard deviations were calculated. Similarly, in order to better interpret the results from the ^86^Rb^+^ uptake assay, data were normalized such that the control condition (no peptide pre-incubation) in each cell type reflected 100% uptake.

## Supporting information

S1 Table Human SST interactome list. The table depicts the SST interactome dataset generated in this study in alphabetical order. The columns depicting iTRAQ ratios reveal the enrichment level of proteins relative to the SST14Δ7–10 negative control, i.e., proteins whose iTRAQ enrichment ratios were observed with values close to 1.0 exhibited no relative enrichment. The ‘Coverage’ column depicts the percentage of primary sequence of a given entry that was covered by peptide-to-spectrum matches (PSMs). The count column indicates the number of PSMs that supported the calculation of values shown in the iTRAQ ratio columns.
(EPS)

## Abbreviations

Aβ: amyloid beta peptide
AD: Alzheimer’s disease
CST: cortistatin
GH: growth hormone
GI: gastrointestinal
GO: gene ontology
GPCR: G protein-coupled receptor
iTRAQ: isobaric tagging for relative and absolute quantitation
PPSST: preprosomatostatin
PSM: peptide-spectrum match
RP: reversed phase
SNAP25: synaptosomal-associated protein 25
SST: somatostatin
SSTR: somatostatin receptor
VIP: vasoactive intestinal peptide
